# Subwavelength grating as both emission mirror and electrical contact for VCSELs in any material system

**DOI:** 10.1038/srep40348

**Published:** 2017-01-12

**Authors:** Tomasz Czyszanowski, Marcin Gebski, Maciej Dems, Michał Wasiak, Robert Sarzała, Krassimir Panajotov

**Affiliations:** 1 Photonics Group, Institute of Physics, Lodz University of Technology, Wólczańska 219, 90-924 Łódź, Poland; 2 Institute of Solid State Physics and Center of Nanophotonics, Technische Universität Berlin, Hardenbergstraße 36, D-10623 Berlin, Federal Republic of Germany; 3 Vrije Universiteit Brussel, Department of Applied Physics and Photonics, Brussels Photonics (B-PHOT), Pleinlaan 2, B-1050 Brussels, Belgium; 4 Institute of Solid State Physics, 1784 Sofia, Bulgaria

## Abstract

Semiconductor-metal subwavelength grating (SMSG) can serve a dual purpose in vertical-cavity surface-emitting lasers (VCSELs), as both optical coupler and current injector. SMSGs provide optical as well as lateral current confinement, eliminating the need for ring contacts and lateral build-in optical and current confinement, allowing their implementation on arbitrarily large surfaces. Using an SMSG as the top mirror enables fabrication of monolithic VCSELs from any type of semiconductor crystal. The construction of VCSELs with SMSGs requires significantly less p-type material, in comparison to conventional VCSELs. In this paper, using a three-dimensional, fully vectorial optical model, we analyse the properties of the stand-alone SMSG in a number of semiconductor materials for a broad range of wavelengths. Integrating the optical model with thermal and electrical numerical models, we then simulate the threshold operation of an exemplary SMSG VCSEL.

One of the major challenges in photonics is to construct monolithically integrated, conductive distributed Bragg reflectors (DBRs) with over 97% optical power reflectance in all material systems used in vertical-cavity surface-emitting laser (VCSEL) technology. Monolithically integrated DBRs, composed of lattice-matched materials in quarter-wavelength pairs of high refractive index contrast layers, can be grown routinely in arsenide-based systems (GaAs and AlGaAs with high aluminium content)^1^. However, they are extremely difficult to fabricate in other material systems, such as GaN- and InP-based materials^2^,^3^,^4^. Alternatives include nonconductive dielectric DBRs^5^ or semiconductor wafer bonded DBRs^6^. However, these types of mirror can degrade current injection to the central part of the active region of VCSELs, below the emission window, requiring use of lateral current confinement methods such as proton implantation^7^, selective wet oxidations^8^ or patterned tunnel junctions^6^. High-refractive-index contrast gratings (HCGs) are another option^9^. These mirrors may be as thin as half the wavelength, which is tens of times thinner than DBRs. HCGs can further provide reflection stop bands twice as broad as those for conventional DBR mirrors, facilitating strong and stable polarization control of emitted light^10^. HCGs can be constructed in several ways^10^,^11^,^12^. However, for close to 100% reflectivity to be achieved in VCSELs, the HCG stripes must be surrounded by low refractive index material.

The low refractive index materials used in HCGs are typically dielectrics (air or oxides), which are insulators. Goeman *et al*.^13^. first proposed using a grating fabricated from a monolithic crystal, achieving power reflectance as high as 85% for polarized light with a wavelength of 1550 nm. In refs 14 and 15, we showed that a monolithic HCG (MHCG) with almost 100% power reflectance could be fabricated from any transparent material with a refractive index greater than 1.75. MHCGs could therefore enable vertical stimulated emission in almost all the most common optoelectronic materials in use today, while significantly reducing the use of expensive and environmentally harmful compounds. Monolithic high-refractive-index contrast gratings integrated with metal contacts, which we call a semiconductor-metal subwavelength grating (SMSG), can serve a dual purpose in VCSELs, as both optical couplers and current injectors.

Our design for a SMSG VCSEL opens new possibilities for fabricating VCSELs with direct current injection, without the need for current or optical lateral confinement, in any semiconductor material system. The main goal of the analysis presented here is to demonstrate, using numerical methods, that an MHCG mirror integrated with metallic contacts (SMSG) can provide enough optical power reflectance to enable stimulated emission. In the next section, we describe the structure of the SMSG mirrors and the VCSEL design considered in the analysis. In section “Power reflectance of stand-alone SMSG mirror”, we analyse the properties of the exemplary GaAs SMSG mirror and the parameters which provide maximal power reflectance. In section “Dispersion in SMSG mirrors”, we examine the influence of refractive index and dispersion on power reflectance, using a range of metals and semiconductor crystals (GaAs, AlGaAs, GaN, InP, Si). In section “VCSEL with SMSG mirror”, we calculate the threshold characteristics of an exemplary 980 nm GaAs-based VCSEL with an SMSG as the top emission mirror.

## Results

**Figure 1:**
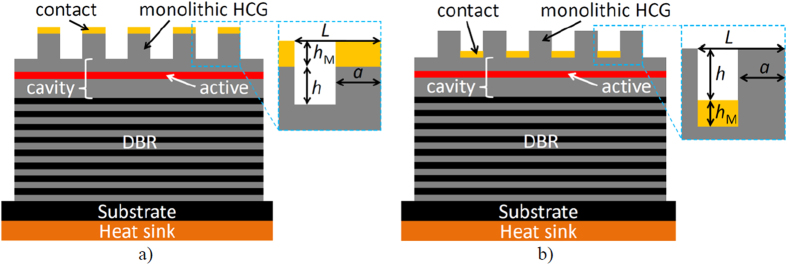
Schematics of VCSELs with SMSGs as top mirrors in the top configuration (**a**) and valley configuration (**b**). Geometrical parameters used in the calculations: *L* is the SMSG period, *a* the width of the stripes, *h* the thickness of the stripes without metal and *h*_M_ the thickness of the metal stripes. The duty cycle of the SMSG is defined as: *F* = *a*/*L*. The bottom contact is placed on the surface below the DBR mirrors.

The stand-alone mirror analysed in section “Power reflectance of stand-alone SMSG mirror” consists of a monolithic GaAs layer with etched stripes. Two variants are considered. In the first, top configuration, gold stripes are deposited on top of the semiconductor stripes (Fig. 1a). In the second, valley configuration, the gold stripes are deposited between the GaAs stripes (Fig. 1b). The thickness of the GaAs and the air beneath the mirror are assumed to be infinite. We consider a single period of the SMSG with periodic boundary conditions^15^, which elongates the mirror periodicity to infinity in the lateral direction. The parameters of the SMSG are as follows: *L* – period of the grating; *h* – height of the stripe below the metal in the top configuration and above the metal in the valley configuration; *h*_*M*_ – thickness of the metal, where *M* is the chemical symbol of the metal; *F* – duty cycle, as the ratio of the width (*a*) over the period (*L*) of the stripe. Unless explicitly stated otherwise, the thickness of the metal is 50 nm. In section “Dispersion in SMSG mirrors”, we will consider only the valley configuration in an exemplary design constructed from various semiconductor materials and metals. The choice of valley configuration is due to its higher power reflectance and higher normalized transmittance with respect to top configuration as it will be shown in section “Power reflectance of stand-alone SMSG mirror”. The assumptions and symbols used in the calculations in section “Dispersion in SMSG mirrors” are the same as in section “Power reflectance of stand-alone SMSG mirror”.

In section “VCSEL with SMSG mirror”, we calculate the threshold characteristics of a VCSEL composed of two mirrors, a GaAs/Au SMSG in the valley configuration and a bottom DBR mirror composed of 35 pairs of GaAs/Al_0.9_Ga_0.1_As quarter-wavelength layers. The second design for a VCSEL with an SMSG in the top configuration is used only to illustrate the conclusions drawn in section III. The current confinement heterostructure (CCH) of the VCSEL is composed of 8 nm In_0.21_Ga_0.79_As quantum wells (QWs) with 6 nm GaAs_0.88_P_0.12_ barriers surrounded by 50 nm Al_0.2_Ga_0.8_As spacer layers. The CCH is sandwiched between phase-matching GaAs layers. The thickness of the top phase-matching layer is tuned for each SMSG configuration separately, to ensure the active region is in the antinode position and that the phase change of the resonant wave during a roundtrip in the cavity is 2π*m* (where *m* is an integer). The epitaxial structure is placed on a conductive substrate mounted on a copper heat sink.

We consider the transverse-electric (TE) mode of light polarisation, in which the SMSG stripes are parallel to the electric field. Transverse-magnetic (TM) polarisation, in which the SMSG stripes are perpendicular to the electric field, is not analysed here. Our calculations reveal very strong light absorption by the metallic stripes in TM mode, with optical power reflectance below 50%, which is insufficient for VCSEL mirrors (data not shown).

### Power reflectance of stand-alone SMSG mirror

Straightforward implementation of gold stripes on the optimal MHCG mirror in ref. 15 reduces its power reflectance drastically. The loss of power reflectance is mostly due to light being absorbed by the gold stripes. However, it is also caused by modified interference conditions between the two grating modes^16^, as the refractive index of air differs significantly from that of gold (which replaces air in SMSGs). In both top and valley configurations, 5 nm thick metallic contacts reduce the power reflectance of the mirror to below 90%. With thickness of gold over 100 nm, power reflectance is reduced to less than 40%. This makes the mirrors unsuitable for VCSEL applications. To maximize the power reflectance of SMSGs, interaction between the metal and the optical field must be minimised. This requires the development of new mirror designs.

**Figure 2:**
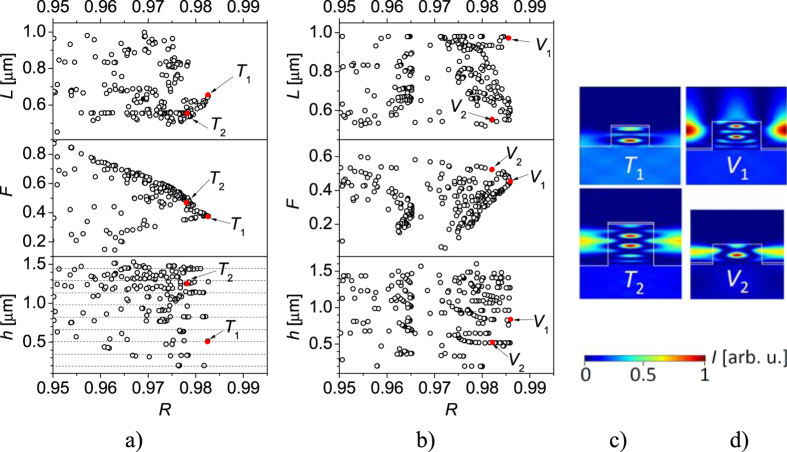
Black dots represent values of LMPRs with respect to their period (*L*), duty cycle (*F*), height (*h*) of the SMSG semiconductor stripes in the case of the top configuration (**a**) and valley configuration (**b**). Red dots indicate designs: *V*_1_ (*L* = 0.972 μm, *F* = 0.484, *h* = 0.762 μm, *R* = 0.985), *V*_2_ (*L* = 0.525 μm, *F* = 0.527, *h* = 0.515 μm, *R* = 0.982), *T*_1_ (*L* = 0.642 μm, *F* = 0.378, *h* = 0.515 μm, *R* = 0.982), *T*_2_ (*L* = 0.564 μm, *F* = 0.446, *h* = 1.25802 μm, *R* = 0.978). Distribution of the optical field intensity within a single period of designs *T*_1_, *T*_2_ (**c**) and *V*_1_, *V*_2_ (**d**).

Using three-dimensional maximization of power reflectance *R*, assuming grating parameters of 0.2 < *L* < 1.2 μm, 0 < *F* < 1 and 0 < *h* < 1.6 μm as variables and constant thickness of gold stripes *h*_Au_ = 50 nm, we found the parameters for SMSGs corresponding to local maxima of power reflectance (LMPR) in the space of grating parameters for top and valley mirror configurations. Figure 2a and b illustrate the distribution of SMSG parameters providing over 95% power reflectance. Some particular tendencies can be noticed. In the SMSG designs with the highest power reflectance, the periods (*L*) are closer to the deep-subwavelength limit. However, several designs in the valley configuration are closer to the diffraction limit (*L *≈ λ and *L* < λ) determined by the limit of two-grating-mode regime^16^, which is of significance for technological implementation due to the largest possible SMSG period. Further increase of SMSG period allows third mode propagation in the grating, which deteriorates the ability of high power reflectance. The maximal value for power reflectance in the top configuration is 98.2%. In the valley configuration, maximal power reflectance is 98.6%. The duty cycles (*F*) of the mirrors with the highest power reflectance are around 0.4 in both configurations. The height of the stripes (*h*) of mirrors with over 98% power reflectance can be arbitrary. However, LMPRs of the highest power reflectance were found with periodical values of *h* corresponding to half the wavelength in the grating, as shown by the dashed lines in Fig. 2a,b.

**Figure 3:**
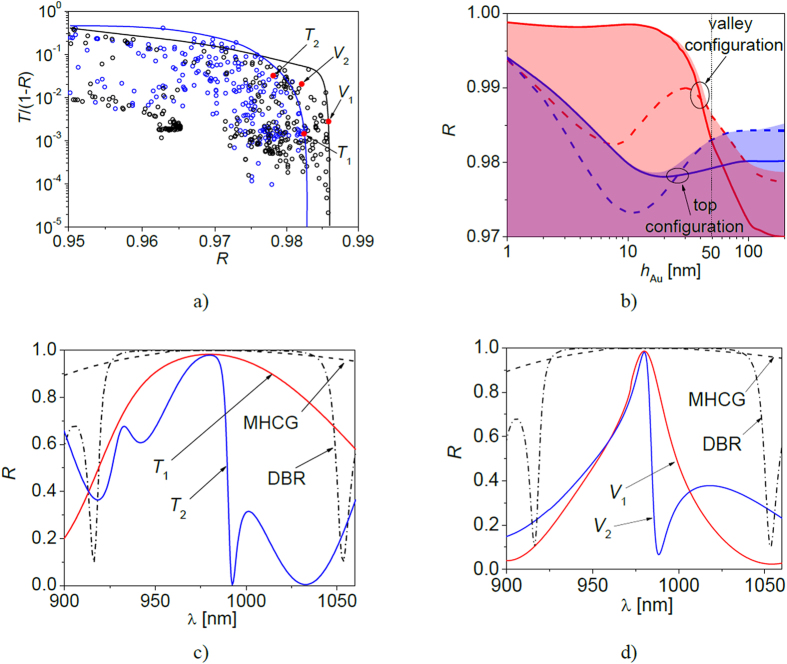
(**a**) Normalized transmittance and corresponding power reflectance of zeroth diffraction order of LMPRs. Blue points correspond to SMSGs in the top configuration and black points to SMSGs in the valley configuration. Red dots relate to selected designs: *T*_1_, *T*_2_, *V*_1_, *V*_2_. Black and blue curves are illustrative boarders of SMSG parameters showing highest power reflectance and the corresponding largest relative power transmittance in valley (black) and top (blue) configurations. (**b**) Dependence of power reflectance of *T*_1_ (dashed blue), *T*_2_ (solid blue), *V*_1_ (dashed red) and *V*_2_ (solid red) mirrors as a function of gold stripe thickness. Fill areas correspond to LMPRs in the top configuration (blue area) and valley configuration (salmon-colored area). Power reflectance spectrum of SMSGs in the top configuration (**c**) and valley configuration (**d**) for chosen SMSG designs. In both figures power reflectance spectra of an MHCG and 35 pair GaAs/Al_0.9_Ga_0.1_As DBR are also shown.

In conventional DBR mirrors, almost all the light which is not reflected is transmitted. Only a small amount of light is absorbed by the DBR layers, due to free carrier absorption. In SMSGs, on the other hand, the use of strongly light-absorbing metal causes severe absorption. Moreover, incident light interacting with the grating is reflected in several diffraction orders, of which only the zeroth order supports lasing. The rest of the light is transmitted in the zeroth diffraction order as useful laser emission^16^. Since SMSGs for VCSEL applications should transmit the maximal amount of unreflected light, we calculated the normalized power transmittance with respect to light which was not reflected in the zeroth diffraction order *T*_n_ = *T/*(1 − *R*), where *T* is absolute power transmittance. Figure 3a illustrates the relative power transmittance of the SMSGs versus zero grating order power reflectance. The most efficient SMSGs are positioned near the blue and red curves. The most efficient designs, with nearly 98% power reflectance, allow for 10% relative power transmittance. The relative transmittance slowly tends towards 50% as power reflectance is reduced to 95%. Figure 3a also shows that, the valley configuration provides more effective light emission than the top configuration close to maximal power reflectance.

We selected two mirrors in each configuration for further analysis (see captions to Figs 2 and 3): two top (*T*_1_, *T*_2_) and two valley (*V*_1_, *V*_2_) configurations. Maximal power reflectance was observed with both 50 nm thick metal layers (*T*_1_, *V*_1_) and very thin (<10 nm) metal layers (*T*_2_, *V*_2_, see discussion of Fig. 3b). In the case of the top configuration, the metal deposited on top of the stripe interacts with and absorbs the transmitted light (Fig. 2c). In the valley configuration, the metal is positioned in the node of the mode and light can be transmitted through the semiconductor-air interface on the tops of the stripes, where there is no metal (Fig. 2d). This enables more efficient light emission and lower absorption in the valley configuration.

Figure 3b shows the power reflectance of the mirrors as a function of the thickness of the gold stripes. Using three-dimensional optimisation, optimal constructions of the SMSGs were found for each *h*_Au_ thickness, with *L, F* and *h* as variables. Curves in Fig. 3b are determined with a thickness step of 1 nm from 1 nm to 50 nm of *h*_Au_ and 10 nm steps from 50 nm to 200 nm of *h*_Au_. The filled areas correspond to the power reflectance values of all other SMSGs considered in this study. In two regions, where 10 nm < *h*_Au_ < 40 nm and *h*_Au_ < 100 nm, the filled area is not below the curves. This corresponds to designs in which other SMSGs are superior. The maximal power reflectance of valley configurations rises to nearly 99.8% as the thickness of the gold stripes is reduced from 50 nm to 20 nm. Further reducing the thickness to 1 nm does not increase power reflectance significantly. Without metal, the power reflectance is nearly 100% and the parameters are the same as those of the MHCG described in ref. 15. Increasing the contact thickness to over 50 nm reduces power reflectance, which falls to 97.9% for *h*_Au_ = 200 nm. In the top configuration, increasing the metal thickness from *h*_Au_ = 1 nm reduces power reflectance, reaching a minimal value of 98% for *h*_Au_ = 20 nm. Further increasing the thickness of the gold stripes increases the power reflectance, which reaches 98.5% for *h*_Au_ = 200 nm. The valley configuration thus has advantages over the top configuration, especially in combination with thin metal stripes. Reducing the thickness of the metal stripes to 20 nm could increase the efficiency of SMSGs to levels comparable to those of standard DBRs. However, in section V we will consider thicker, 50 nm metal stripes, to demonstrate that not only the SMSG with the most favourable parameters enables reaching the lasing threshold in VCSELs.

Figure 3c,d show the power reflectance spectra of the SMSGs in top (Fig. 1a) and valley (Fig. 1b) configurations, together with the spectra of a GaAs MHCG and a DRB with 35 GaAs/AlGaAs pairs. Both configurations of SMSG reveal significantly narrower reflection spectra than those of the conventional DBR and MHCG. These narrow spectra imply the need for high precision in the fabrication of SMSGs, as there is a 1% reduction in power reflectance for each 3.5 nm imprecision in their spatial dimensions. Reducing the thickness of the metal stripes in the SMSG broadens the reflection spectrum, which transforms into an MHCG spectrum as the thickness of the metal stripes tends towards 0. Since the top configuration provides less efficient power reflectance and lower normalised power transmittance, we will consider only the valley configuration in the next section.

### Dispersion in SMSG mirrors

Dispersion is a complex problem in SMSGs since the refractive indices of the metals and semiconductors change in a nonlinear manner with the wavelength. This requires a modification of SMSG geometry to sustain high power reflectance. We will begin with an analysis of the refractive indices of mirror without considering dispersion. The metal stripes now will be called “filler” since its analysed broad range of refractive index values exceeds those for real-world metals. We analyse the influence of the filler complex refractive index and semiconductor real refractive index on power reflectance of SMSG. All the results presented in this section correspond to the LMPRs, according to three-dimensional SMSG parameter optimisation in the space of *L, F* and *h*.

**Figure 4:**
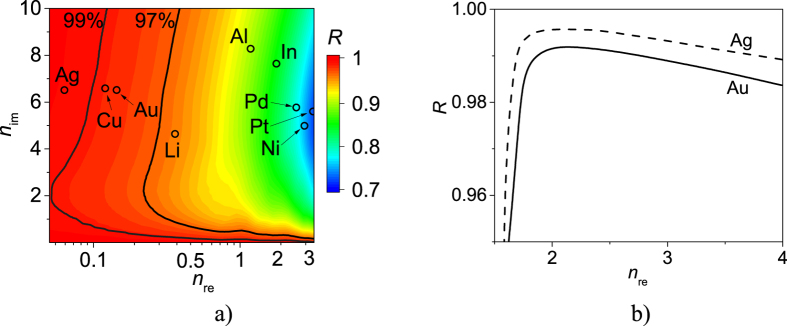
Power reflectance of *V*_1_ at 980 nm as the function (**a**) of real (*n*_re_) and imaginary (*n*_im_) parts of the metal refractive index and (**b**) the refractive index of the semiconductor with gold and silver stripes. Refractive indices of selected metals^17^,^18^,^19^,^20^,^21^ are indicated in (**a**).

Figure 4a illustrates the influence of the complex refractive index (*n*_re_ – real and *n*_im_ – imaginary component) of 50 nm thick filler on the power reflectance of *V*_1_ configuration. Although the map in Fig. 4a was calculated based on the SMSG with the highest power reflectance, qualitatively similar power reflectance maps can be plotted for other designs. A common feature of the maps for various SMSG designs is their high power reflectance, which can be achieved for two cases: first, for an arbitrary real refractive index of the filler and its nearly zero imaginary part, which corresponds to the case of an MHCG and second for the arbitrary imaginary part of the refractive index of the filler and its real part below 0.5.

Of the metals included in the analysis, thin layers of silver, copper and gold have the lowest *n*_re_, at 980 nm, and as a consequence provide the highest power reflectance. Reducing the thickness of the filler shifts the region of >99% reflection toward higher *n*_re_, allowing other metals to be used in SMSGs. Moreover optical properties of thin metal layers are also related to the technology of metal implementation^19^ and can be the subject of further works aimed for lowering their real refractive index. Figure 4b illustrates the dependence of power reflectance as a function of the refractive index of semiconductors with metallic stripes made of either silver or gold. In each of the two cases, the power reflectance increases as the refractive index of the semiconductor decreases from 4 to 2. If the semiconductor refractive index decreases the electromagnetic wave corresponding to LMPR becomes longer in semiconductor. Hence, the size of the metallic stripes effectively becomes smaller with respect to the wavelength, which in turn reduces interaction between the metallic stripes and the optical field. It follows that reducing the refractive index of an SMSG semiconductor will also reduce absorption in the metallic stripes. Reducing the refractive index of an SMSG so that *n* < 2 produces a low refractive index contrast between the semiconductor and air^14^,^15^. This prevents the two grating modes from being guided and prevents from their destructive interference, which enables high power reflectance^16^. The analysis presented here therefore suggests that lower refractive index materials are preferable for use in SMSGs. Given that the grating stripes in valley configurations are undoped, they could be replaced by alternative nonconductive materials with different refractive indices, while the rest of the SMSG construction would remain unchanged, further increasing the efficiency of the SMSGs.

**Figure 5:**
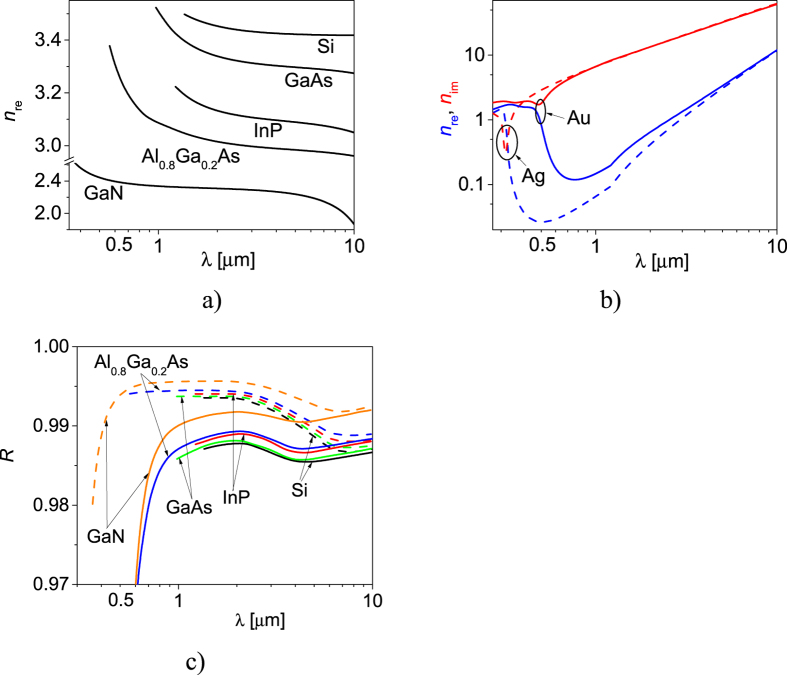
Refractive indices of chosen semiconductors^22^,^23^,^24^,^25^,^26^ (**a**) and metals^17^ (**b**) versus wavelength. Maximal power reflectance versus wavelength for SMSGs composed of selected semiconductors with gold stripes (solid curves) and silver stripes (dashed lines) (**c**).

As shown in Fig. 4b, SMSGs can be constructed from any material with a refractive index greater than 1.75^14^ and low absorption. We chose to focus on semiconductor crystals (GaN, Al_0.8_Ga_0.2_As, GaAs, InP, Si) used in VCSELs with emission from ultraviolet to infrared. Figure 5a illustrates the dispersion of these semiconductor materials in this wavelength range from the absorption edge to 10 μm. Figure 5b illustrates the analogous dispersion of gold and silver. Figure 5c shows the spectral characteristics of SMSGs composed of the semiconductor materials with 50 nm thick silver (dashed curves) or gold (solid curves) stripes. The results confirm that combinations of metals with lower real refractive indices and semiconductors with real refractive indices close to 2 provide the highest power reflectance. In Table 1 exemplary designs of SMSGs have been collected, which relate to the most important applications of VCSELs in the visible spectral range as red, green and blue light emitters and in telecommunication windows. VCSELs for those wavelengths are facing great difficulties due to the lack of conductive, monolithically integrated DBRs, which could provide high power reflectance and moderate number of quarterwavelength layers. The only two exceptions are GaAs/AlGaAs DBRs for 850 and 980 nm wavelengths. The designs collected in the table relate to maximal power reflectance achieved for relatively small etching depth of stripes (*h*) and large period (*L*) which facilitates their fabrication.

The results presented in this section are an idealisation and do not consider fabrication tolerance. The real-world implementation of SMSG mirrors requires optimisation of the contact technology. This includes ensuring the mechanical stability of the metallic stripes, selecting metal alloys with low real refractive indices and fabricating a high quality metal-semiconductor junction. The narrow stop-band in SMSGs presents another technological challenge, due to the high yield required in the fabrication process. Nevertheless, the role of numerical analysis is not only to explain physical processes in real-world devices but also to prefigure possible future devices and concepts. Thus, in the next section we will consider a novel current injection mechanism in VCSELs, where the SMSG plays a double purpose as both an electric contact and a highly reflective mirror.

### VCSEL with SMSG mirror

The power reflectance of DBRs used in modern VCSELs, through which light is emitted, is around 99.8%. This provides a balance between low threshold current and high emitted power^27^ or a high modulation bandwidth^28^. However, the emitted power of a single VCSEL is limited by the current crowding effect. This occurs due to the requirement for at least one ring contact^29^, which restricts the size of the active region. Semiconductor metal gratings could reduce the effect of current crowding in VCSELs, since neither ring contacts nor current confinement mechanisms are needed. Moreover, SMSGs allow for large lateral active regions, by simply scaling the SMSG. A significant disadvantage of SMSGs in comparison to conventional DBRs is their lower emission efficiency, due to their intrinsic absorption. However, the ability to arbitrarily scale their size and the possibility of fabricating SMSGs from any semiconductor material are significant advantages, which are expected to compensate for their lower efficiency.

**Figure 6:**
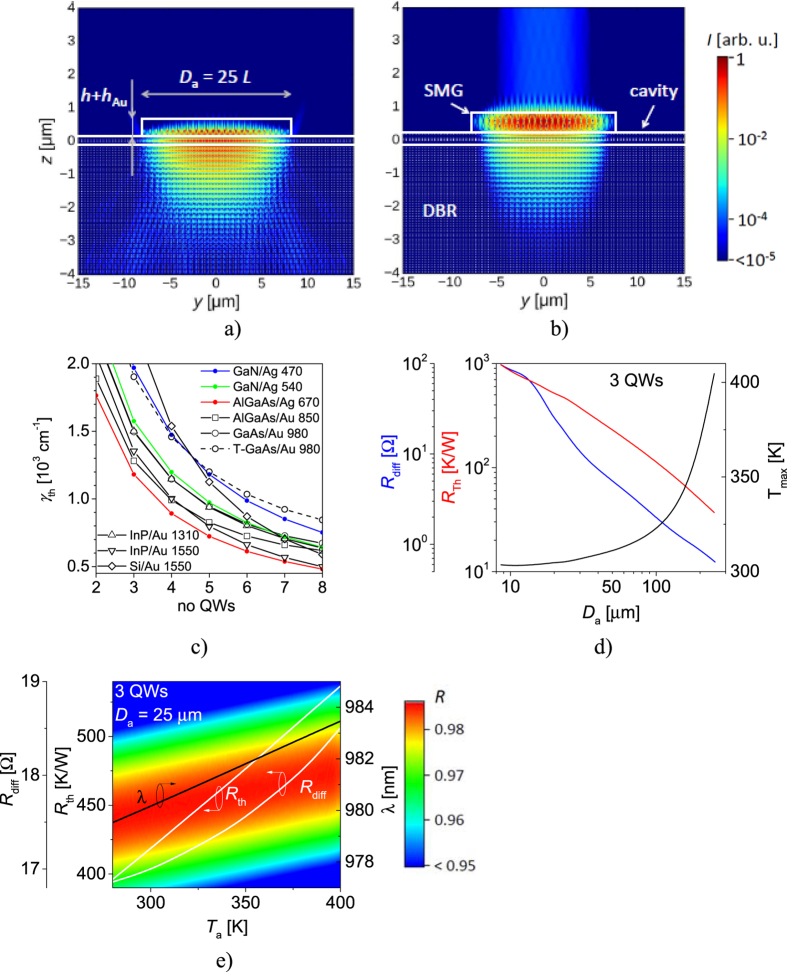
Intensity of fundamental (HE_11_) SMSG VCSEL mode in the plane perpendicular to SMSG stripes in the top configuration (**a**) and valley configuration (**b**). The color scale corresponds to the mode intensity on a logarithmic scale. Solid lines show the borders of the SMSG and the cavity. SMSG is composed of 19 periods of parameters: *L* = 0.972 μm, *F* = 0.484, *h* = 0.762 μm (*V*_1_) and three side periods on both ends of modified duty cycle *F* = 0.43. (**c**) Threshold material gain as a function of the number of quantum wells in SMSG VCSELs in the selected designs from Table 1, with 41 SMSG periods, T-GaAs/Au corresponds to design *T*_1_; differential resistance (*R*_diff_) and thermal impedance (*R*_th_) of the SMSG VCSEL in the valley configuration as functions of (**d**) the size of a square-shaped SMSG (*D*_a_); and (**e**) the ambient temperature (*T*_a_). Maximal temperature (*T*_max_) within the active region in (**d**) and resonant wavelength in (**e**) are indicated by black curves. Color map in (**e**) presents the power reflectance spectrum of *V*_1_ configuration as a function of ambient temperature.

In the previous section, we considered a stand-alone SMSG mirror composed of a single period with periodic boundary conditions. In this section, we will perform a full simulation of SMSG VCSEL (Fig. 1a,b) with a finite number of MHCG periods. The last three outer SMSG stripes are given different duty cycles, providing lateral optical confinement, as proposed in ref. 14. Figure 6a,b illustrate the distributions of the logarithm of mode intensities in the plane perpendicular to the SMSG stripes (25 periods), in top (Fig. 6a) and valley (Fig. 6b) configurations. In both VCSEL configurations, the optical field is located predominantly in the vicinity of the SMSG stripes and the cavity. As anticipated in our analysis of the stand-alone mirror, the main difference between the designs is the significant top-vertical emission that occurs in the valley configuration. There is almost no top-vertical emission in the top configuration. Another difference concerns light scattering in the bottom direction from the higher diffraction orders, which are not fully dumped in the top configuration. The phase of these higher diffraction orders is different from that of the zero-order reflected beam, to which the thickness of the cavity is tuned. Scattering does not occur in the valley configuration.

The lower reflectivity of SMSG means that SMSG VCSELs have higher threshold currents than conventional VCSELs. To achieve an acceptable level of threshold material gain per single quantum well, the number of quantum wells must therefore be increased, while keeping the center of the active region in an antinode of the standing wave and adequately redesigning the cavity to sustain resonant wavelength at 980 nm.

In the valley configuration, since there is no current flow in the region of the semiconductor stripes, the semiconductor stripes were assumed to be undoped. Figure 6c shows the dependence of the averaged (over the area of the SMSG) material threshold gain (*γ*_th_) per single quantum well for selected designs from Table 1. The lateral gain distribution was calculated taking into account carrier diffusion in the active region. The current was injected through a square SMSG with an edge length of 25.3 μm (41 periods). Concerning SMSG VCSEL designed for 980 nm wavelength emission in the valley configuration (black solid line, open circle), a level of threshold gain <1500 cm^−1^ was achieved with three 8 nm In_0.2_Ga_0.8_As quantum wells. The same VCSEL with SMSG in the top configuration (black dashed line, open circle), *γ*_th_ was typically 25% higher, which can be explained on one hand by the lower power reflectance of SMSGs, but also by absorption occurring in the semiconductor stripes (located in the thin layer below the metal stripes). Increasing the number of quantum wells to over six reduces *γ*_th_ to less than 800 cm^−1^ in the valley configuration, which is a typical level for the threshold gain per quantum well of a conventional VCSEL. Figure 6c also illustrates threshold gain of VCSELs incorporating mirrors collected in Table 1. For each VCSEL designed for particular emission wavelength one mirror was selected with silver or gold stripes. Mirrors with 50 nm thick gold stripes for emission in visible range reveal low power reflectance precluding low threshold gains. Therefore mirrors with silver stripes were selected for VCSELs emitting in visible range. In the infrared range, 50 nm thick gold stripes provide also lower SMSG power reflectance with respect to their counterparts with silver stripes, but sufficiently high to achieve acceptable level of the threshold gain. Therefore mirrors with gold stripes are considered in infrared range. Exemplary quantum wells have been embedded in the following VCSEL cavities: 3 nm In_0.18_Ga_0.82_N at 470 nm, 3 nm In_0.22_Ga_0.78_N at 540 nm, 4.4 nm In_0.57_Ga_0.43_P at 680 nm, 10 nm In_0.08_Ga_0.92_As at 850 nm and 8 nm InAlGaAs at 1330 nm and 1550 nm. All mirrors are monolithically integrated with VCSEL cavities but Si/Au mirror which is fused to InP cavity such that interface between Si and InP coincides with the antinode of the standing wave. The threshold gain of analysed VCSELs is not a function of the SMSG power reflectance only but of the confinement factor of the active region as well. The confinement factor depends on the active region thickness relative to the wavelength in the active region. That promotes thick quantum wells of high refractive index, as for example InGaAs, and handicaps InGaN quantum wells which are of low refractive index.

Figure 6d shows the threshold characteristics of an 980 nm SMSG VCSEL in the valley configuration, as a function of the lateral dimensions of a square SMSG. Increasing the aperture size reduces differential resistance (*R*_diff_) and thermal impedance (*R*_th_). This leads to widening of the current path, as well as to improved heat extraction from the broader surface of the active region. However, increasing the aperture contributes to raising the threshold maximal temperature in the active region. The temperature increase becomes abrupt for the size of the square-shaped SMSG aperture (*D*_a_) over 200 μm, as more electrical power is injected into devices with larger SMSGs. If we compare the smallest device considered here (*D*_a_ = 8.5 μm) to the state-of-the-art conventional VCSELs with similar dimensions^30^ to which our model was calibrated^31^,^32^, a significant difference can be observed in terms of threshold electrical power, which is 2.5 times greater (3.7 mW) in the case of the SMSG VCSEL. However, the differential resistances are comparable, since the same junctions are used in both cases. Thermal impedance is half that of the conventional VCSEL. This is related to the elimination of the p-type DBR through which current is injected in conventional VCSELs.

Figure 6e shows the threshold characteristics of the 980 nm SMSG VCSEL design as a function of the ambient temperature. The loss of electrical and thermal performance as the ambient temperature rises is due to the shift in the gain spectrum towards longer wavelengths (dλ/d*T* = 0.39 nm/K), which is not followed by a shift in the resonant wavelength (dλ/d*T* = 0.03 nm/K). It is also explained by reductions in the electrical and thermal conductivities of the device. A change in ambient temperature from 280 K to 400 K causes an increase in threshold power from 11 mW to 27 mW in a device of *D*_a_ = 25 μm. The narrow power reflectance spectrum of SMSG would suggest very small operational temperature range. Assuming that the design had stable power reflectance spectrum while temperature is changing, the reduction of power reflectance to below 0.97 for resonant wavelength could occur at 375 K. However temperature changes in the device induce shift in the SMSG power reflectance equal to dλ/d*T* = 0.012 nm/K, with an insignificant reduction in the maximal power reflectance (see color map in Fig. 6e). Simultaneous thermal shifts in the resonant wavelength and power reflectance spectrum enable lasing at elevated temperatures up to 425 K for which the power reflectance of considered SMSG drops below 0.97. The analysed SMSG VCSEL is designed to enable minimal threshold gain at 300 K of ambient temperature. However the operational temperature of the laser can be increased by shortening the cavity so as to allow overlapping of the resonant wavelength and the maximal power reflectance of SMSG at higher temperatures. More stable thermal behaviour of the device could be achieved at the cost of higher threshold gain at 300 K of ambient temperature.

## Discussion

This paper has proposed a design for a new VCSEL structure, in which the top distributed Bragg reflector is replaced by an SMSG. In our novel design, this grating serves a dual purpose, as both an optical coupler and the current injector. By a numerical analysis, we showed that a mirror in the form of subwavelength grating with metallic stripes, etched in monolithic crystal, can provide optical power reflectance >97% for semiconductor materials with a refractive index over 1.75, when the real refractive index of the metallic stripes is below 0.5. We investigated two SMSG configurations, in which the metal contacts were implemented on top of the subwavelength stripes (top configuration) or between the subwavelength stripes (valley configuration). The valley configuration showed higher power reflectance and greater efficiency in terms of light transmitance, due to reduced interaction between the optical field and the metal in comparison to the top configuration. However, the efficiency of optical power emission in the valley configuration was still a tenth of that achieved by DBRs.

We next simulated the threshold operation of various VCSELs with an SMSG as the top mirror. Our analysis shows that the valley configuration enables top emission through the SMSG. Although the efficiency of SMSGs is lower than that of conventional DBRs, the new SMSG VCSEL structures reach threshold gain below 1500 cm^−1^ for all considered VCSEL designs emitting from visible range to infrared. The thermal and electrical properties of the SMSG VCSEL are comparable to or surpass those of conventional VCSELs. This is largely attributable to the possibility for direct current injection into the active region and reduced use of p-type material. The current crowding effect is also minimized in SMSGs, permitting nearly uniform carrier injection even for large devices over the entire active region. Although SMSGs have very narrow power reflectance spectra, the structure reached threshold even when the temperature in the active region exceeded 400 K. The reason for the substantial increase in laser threshold gain was the detuning between the resonant wavelength and the power reflectance spectrum of the SMSG.

Further studies of SMSG VCSELs should focus on the composition of the top-contact. Since the top-contact is placed close to the optical field maximum in SMSG VCSELs, it has a strong influence over the efficiency of the device. In our analysis, uniform gold or silver 50 nm contacts were assumed. However, a metallic interface layer between the gold or silver contacts and the semiconductor should be used to reduce the Schottky barrier, as this can affect optical power reflectance. A thinner contact of less than 50 nm could also be used, to increase the power reflectance and broaden the reflection spectrum.

The new perspectives opened by SMSG VCSELs include the possibility of using any semiconductor material system for their fabrication, such as phosphide-, nitride- or zinc-based materials, which have already proven their effectiveness in diode lasers. The need for p-type DBRs is eliminated, as is the requirement for optical and current confinement, which can be achieved by manipulating the parameters of the SMSG during the processing stage. SMSG implemented on both sides of the cavity enables entire elimination of DBRs^13^. Moreover, SMSG VCSELs retain the possibility of wavelength control via the subwavelength grating, as demonstrated in HCG VCSELs^33^, allowing the fabrication of VCSEL arrays capable of emitting various wavelengths.

## Methods

To simulate the physical phenomena taking place in SMSGs and an SMSG VCSEL, we used three-dimensional models of optical phenomena (based on the Plane Wave Admittance Method - PWAM)^34^. In section “VCSEL with SMSG mirror”, these three-dimensional models were combined with a gain model and a self-consistent model including thermal, electrical and diffusion phenomena (all three based on the Finite Element Method)^35^. This comprehensive model enables precise observation of mode modifications under different lasing conditions. The model has previously revealed good agreement with experimental results in refs 36 and 37, and in particular for a 980 nm VCSEL with the similar cavity and bottom DBR^31^,^32^ to that analysed here. Our model has furthermore shown very good agreement with experimental measurements for power reflectance in a 980 nm wavelength GaAs MHCG^15^. The most important optical and thermal material parameters used in the analysis are given in Table 2.

To determine the power reflectance of the stand-alone SMSG mirror, we took into account a single SMSG period combined with periodic boundary conditions. Thirty plane waves were used in the optical model to reach convergence. In our calculations of the power reflectance (*R*) and power transmittance (*T*), we considered the reflected and transmitted zero grating order, which is perpendicular to the plane of the SMSG.

Local maxima of power reflectance (LMPR) are found using PWAM algorithm calculating the power reflectance for given stand-alone SMSG structure. Varying the geometrical parameters of SMSG: *L* – period; *F* – duty cycle and *h* – height of the SMSG stripe are varied while keeping all other parameters of SMSG constant. Initial search of LMPRs is carried out in the parameters range: 0.2 < *L*/λ < 1.2, 0 < *F* < 1 and 0 < *h*/λ < 1.6 with steps of: Δ*L*/λ = 0.005, Δ*F* = 0.05 and Δ*h*/λ = 0.01. The initial search provides the set of starting parameters (*L, F, h*) for more accurate search of the power reflectance maxima, which is performed with multidimensional Nelder-Mead simplex algorithm^38^.

In the optical simulation of the VCSEL, we considered several numbers of SMSG periods with absorbing boundary conditions (ABC). The calculation window for the optical model was twice the total lateral size of the SMSG, to ensure light decay before ABC. This configuration required 2·30·*n*_s_ plane-waves (*n*_s_ - number of SMSG stripes) in the lateral direction perpendicular to the stripes, making the calculations extremely demanding in terms of time and computer memory. Since calculations for a finite SMSG VCSEL would have required 50–100 times the number of planewaves, making the calculations even more demanding, we limited the optical simulations to only 41 SMSG periods. Thus, we have provided a full physical analysis of <26 μm square aperture VCSELs, and for larger devices we assume that the averaged threshold material gain in the active region is the same as in the case of an SMSG with 41 periods and self-consistent calculations were performed taking into account electrical, thermal diffusion and gain models.

## Additional Information

**How to cite this article:** Czyszanowski, T. *et al*. Subwavelength grating as both emission mirror and electrical contact for VCSELs in any material system. *Sci. Rep.* **7**, 40348; doi: 10.1038/srep40348 (2017).

**Publisher's note:** Springer Nature remains neutral with regard to jurisdictional claims in published maps and institutional affiliations.

## Figures and Tables

**Table 1:** Exemplary designs (*L, F, h*) of SMSGs in valley configuration and their power reflectance corresponding to blue, green, red light and telecommunication wavelengths.

grating	metal	wavelength [μm]	*L* [μm]	*F*	*h* [μm]	*R*
GaN	Ag	470	0.4597	0.4630	0.1271	0.9933
GaN	Ag	540	0.5252	0.4663	0.1546	0.9946
Al_0.8_Ga_0.2_As	Au	680	0.4642	0.4264	0.3891	0.9775
	Ag		0.4559	0.4375	0.3879	0.9940
Al_0.8_Ga_0.2_As	Au	850	0.5836	0.4356	0.5105	0.9851
	Ag		0.6011	0.4428	0.9479	0.9943
GaAs	Au	980	0.9722	0.4835	0.7621	0.9855
	Ag		0.9725	0.4829	0.7622	0.9935
InP	Au	1310	0.8775	0.4390	0.7761	0.9877
	Ag		0.8669	0.4456	0.7762	0.9940
InP	Au	1550	1.0571	0.4313	0.9351	0.9886
	Ag		1.0549	0.4320	0.9362	0.9938
Si	Au	1550	0.9399	0.4634	0.8457	0.9877
	Ag		0.9416	0.4623	0.8459	0.9935

SMSGs are composed of selected semiconductors (typically used in light emitters designed for those wavelengths) and gold or silver stripes.

**Table 2:** Optical, electrical and thermal parameters of 980 nm SMSG VCSEL layers used in the simulations.

Layer	Refractive index at 980 nm	Thermal conductivity [W/(mK)]
Air	1.0	2.5 10^−2^
Au	0.144–6.52i	317
Al_0.98_Ga_0.02_As	2.943	57
Al_0.8_Ga_0.2_As	3.047	16
GaAs	3.521	45
GaAs_0.88_P_0.12_	3.5	30
In_0.21_Ga_0.79_As	3.7	10

The ranges of electrical conductivity correspond to different doping types and levels.
